# University of Wisconsin

**Published:** 1867-08

**Authors:** 


### *University of Wisconsin.*

Institutions of learning, of whatever character, which are subject to political control are likewise subject to rapid and often unexpected mutations. No position is safe from attack, and no weight of scientific or personal reputation will protect from assault. Members of their Faculties have to descend to the dirty work of politics, or, worse still, toady to temporary rulers who hold the places at their will. Nepotism, and back-stairs and scullery management are likely to be tenfold more efficacious, in securing and retaining professorships, than real worth and great abilities. Hence, Faculties are liable to become asylums for seedy political hangers-on, or light-witted brothers-in-law, nephews, or cousins. The University of Michigan, in past years, has been an apt illustration of this truth—we should say more, but the subject is too vast—we feel too vividly the *embarrasse du richesse.*

The University of Wisconsin is passing through a similar career in the wilderness. Its present Board of Regents are wiser in their generation than the husbandman of the old parable. They are rooting out tares and wheat both together. In scraping off the barnacles which obviously impeded the progress of their University craft, they are, we fear, scraping off also sheathing, and planks, and knees. Make haste slowly—venerable Literary Fathers!

When we read in the papers that, under any pretence, such men as Prof. EZRA L. CARR are to be compelled to leave professorships which it is positive condescension for them to consent to occupy, we are forced to believe there is "something rotten in"—the University of Wisconsin. It is unnecessary to say that Prof. Carr has a national reputation, and would be gladly selected to fill any vacancy which might occur in the oldest and proudest literary or scientific institutions in the land. His lectures are marvels of compact learning, expressed in the most forcible and eloquent method. But we did not set out to write an eulogium upon Prof. Carr, nor will we do so. We only wish, as one of the profession of which he is a distinguished ornament, to enter an earnest protest against any such desecration of temporary power.

We are aware of the pitiful expedients usually resorted to by men about to perpetrate such acts—of the village gossip that is retailed. Mrs. Grundy and Paul Pry are at these times unusually busy, but Prof. Carr's position is too high and his reputation too well established to be injured by the Joseph Surfaces and Aminadab Sleeks of society. A personal acquaintance of over a score of years of active professional life, enables us to speak understandingly of the high and deserved reputation of this most estimable gentleman.

It is sincerely to be hoped that the newspapers are wrong, and that no such suicidal act is contemplated.

